# Chemical Antioxidant Quality Markers of *Chrysanthemum morifolium* Using a Spectrum-Effect Approach

**DOI:** 10.3389/fphar.2022.809482

**Published:** 2022-02-07

**Authors:** Yi-Fan Lu, Ding-Xiang Li, Ran Zhang, Lin-Lin Zhao, Zhen Qiu, Yan Du, Shuai Ji, Dao-Quan Tang

**Affiliations:** ^1^ The Second Clinical College, Xuzhou Medical University, Xuzhou, China; ^2^ Jiangsu Key Laboratory of New Drug Research and Clinical Pharmacy, Xuzhou Medical University, Xuzhou, China; ^3^ Department of Pharmaceutical Analysis, Xuzhou Medical University, Xuzhou, China

**Keywords:** Q-marker, *Chrysanthemum* morifolium cv., chromatographic fingerprint, antioxidant activity, spectrum-effect relationship

## Abstract

Traditionally, the quality evaluation of *Chrysanthemum morifolium* (CM) cv. (Juhua) attributes its habitats and processing methods, however, this strategy of neglecting bioactive ingredients usually results in deviation of quality evaluation. This study aims to explore the quality marker (Q-marker) based on spectrum-effect relationship and quality control strategy of CMs. The chromatographic fingerprint of 30 flower head samples of CMs from five different habitats including Hang-baiju, Gongju, Huaiju, Taiju and Boju were constructed by high performance liquid chromatography and analyzed through chemometrics methods such as similarity analysis (SA), cluster analysis (CA) and principal component analysis (PCA). The common peaks were quantified by external standard method and relative correction factor method. The *in-vitro* radical scavenging capacity assays of DPPH·, ·OH and ABTS were carried out. The Q-marker was explored by the correlation analysis between the contents of common peaks and *in-vitro* radical scavenging capacity, and then used to evaluate the quality of 30 flower head samples of CMs. A total of eight common peaks were appointed in 30 flower head samples of CMs, and their similarities ranged from 0.640 to 0.956. CA results showed that 30 flower head samples of CMs could be divided into five categories with reference to the Euclidean distance of 5. PCA results showed that common peaks played a major role in differential contribution of CMs. The quantification of common peaks hinted that their contents possessed significant variation whether for different accessions or the same accessions of CMs. The correlation analysis showed that chlorogenic acid, 3,5-*O*-dicaffeoylquinic acid, unknown peak 1, 4,5-*O*-dicaffeoylquinic acid and kaempferol-3-*O*-rutinoside could be used as the Q-markers for the quality evaluation of 30 flower head samples of commercially available CMs. The analysis strategy that combines chromatographic fingerprint analysis, multiple ingredients quantification, *in-vitro* chemical anti-oxidant activity evaluation and spectrum-effect relationship analysis clarified the therapeutic material basis and discovered the Q-markers, which possibly offers a more comprehensive quality assessment of CMs.

## Introduction

*Chrysanthemum morifolium* (CM) cv. (Juhua) is the dried flower head of *Chrysanthemum morifolium* (Ramat.) Hemsl., and has the effects of dispersing wind and clearing heat, calming the liver and clearing the eyes, and clearing heat and detoxifying the body (Tu et al., 2021; Yang et al., 2019). The *Compendium of Materia Medica* records that CM can benefit the five veins, regulate the extremities, and cure the head wind heat tonic. Modern pharmacological researches also show that CM possesses heat dissipation, detoxification, brightening eyes, lowering blood pressure and other effects (Hodaei et al., 2021a). The main active ingredients of CM include flavonoids (luteolin), phenolic acids (chlorogenic acid), and polysaccharides (Hodaei et al., 2018; Tian et al., 2020), and *Chrysanthemum* extracts containing different components have the ability to improve myocardial nutrition, remove reactive oxygen radicals, strengthen vascular resistance, and lower blood lipids (Zhang et al., 2019). For instance, phenolic acids extract has antibacterial, antiviral, anti-infective, and anti-inflammatory effects (Hodaei et al., 2021b; Youssef et al., 2020; Cho et al., 2021), while polysaccharides extract has anti-tumor and immunity enhancing effects (Jing et al., 2016). Among these effects, antioxidant capacity is the most important pharmacological efficacy of CM and the basis for the treatment of different diseases (Bai et al., 2018; Liu et al., 2018; Lin et al., 2010). The pharmaceutical value and safety of CM have enabled it to be broadly applied as a homogeneous medicinal herb for medical and edible purposes.

There are about 18 species of CMs in China, including 11 medicinal species, mainly cultivated in Zhejiang, Anhui, Henan and other provinces. Currently, about nine species of CMs are used in the market, such as Boju, Chuju, Gongju, Huaiju, Hangju, Taiju, Qiju, Jiju, and Chuanju, and five of them including Boju, Huaiju, Gongju, Hangju, and Chuju are recorded in the *Chinese Pharmacopoeia* (Ch.P.), according to their origins and processing methods. Moreover, there are a variety of wild CMs used in the clinical and food fields. The diversity of species, habitat, cultivation pattern and processing method results in the quality variation of CMs, effective quality control has thus become an important guarantee for the application of CMs in the medical and food fields (Song et al., 2018).

At present, the quality control strategies of CM mainly include multi-ingredients quantification, chromatographic fingerprint and/or their combination. For example, quantification of chlorogenic acid (ChA), luteolin-7-O-glucoside (L-7G), and 3,5-*O*-dicaffeoylquinic acid (3,5-DCQA) and chromatographic fingerprint analysis were respectively used to control the quality of CMs by Ch.P. or other scholars (He et al., 2021; Dai and Sun, 2021). These strategies have provided an assurance for the quality of CMs to a certain extent. Recently, the concept of quality marker (Q-marker) has provided a new idea for the quality control of traditional Chinese medicine (TCM) (Tian et al., 2019), which is based on the perspective of biological activity, and through a variety of ways to find the chemical components that can reflect biological effect of TCM (Chen et al., 2021). Among them, the strategy to explore Q-marker from the perspective of the correlation between the chromatographic spectrums and biological effect (spectrum-effect) has been widely used in the study of various TCMs (Chen L. et al., 2020; Li et al., 2020; Jiang et al., 2021). A predictive analysis on the Q-marker of CMs was conducted by the review of its chemical composition and pharmacological effects (Zhou et al., 2019), however, the study to explore the Q-marker for the quality control of CM from the aspect of spectrum-effect relation has not been published to now.

Antioxidant activity of CMs is the important basis for its edible and medicinal application. Hence, based on chemical antioxidant activity, this study aimed to explore the Q-marker of CMs from the aspect of spectrum-effect relation. Chromatographic fingerprint analysis of 30 flower head samples of five accessions of CMs was firstly performed by high performance liquid chromatography (HPLC) to find common peaks, and chemometric analysis such as similarity analysis (SA), cluster analysis (CA), and principal component analysis (PCA) were used to evaluate the difference of 30 flower head samples of CMs. Common peaks were quantified by external standard method and relative correction factor method. Meanwhile, the chemical antioxidant activities of 30 batches of CMs were evaluated by *in-vitro* free radical scavenging activity such as DPPH, OH and ABTS. The correlation analysis between the common peak and the chemical antioxidant activity was further performed to explore the Q-marker of CM, and the Q-marker was then validated by evaluating the quality of commercially available CMs.

## Materials and Methods

### Materials and Reagents

Methanol and formic acid of chromatographic grade were purchased from Beijing Mreda technology Co., Ltd. (Beijing, China). The reference standards including L-7G, 3,5-DCQA, 4,5-*O*-dicaffeoylquinic acid (4,5-DCQA), apigenin-7-*O*-glucuronide (A-7G), and kaempferol-3-*O*-rutinoside (K-3R) were purchased from Sichuan Weikeqi Biological Technology Co., Ltd. (Chengdu, China), while ChA was purchased from Chengdu Purechem-standard Biological Technology Co., Ltd. (Chengdu, China). Their purities were all not less than 98%. Potassium persulfate, salicylic acid, 1,1-Diphenyl-2-picrylhydrazyl (DPPH), and 2,2′-biazo-bis-3-ethylbenzothiazoline-6-sulfonic acid (ABTS) of analytical grade were purchased from Shanghai Maclean Biochemical Technology Co., Ltd. (Shanghai, China), while ferrous sulfate heptahydrate was purchased from Shanghai Aladdin Biochemical Technology Co., Ltd. (Shanghai, China). Other reagents were of analytical grade and water was purified using a Milli-Q system (Millipore, Bedford, MA, United States).

A total of 30 flower head samples of CMs were collected and identified by Associate Professor Shuai Ji of our laboratory as the dried flower heads of *Chrysanthemum morifolium* (Ramat.) Hemsl. The information of the samples was shown in Table 1.

**TABLE 1:** Information of 30 batches of CM samples.

No.	Accessions	Lot No.	Manufacturer	Place of production
JH-01	Gongju	191208	Bulk packaging in Xuzhou Deren Clinic	Huangshan, Anhui
JH-02	Gongju	20200621	Purchased online	Huangshan, Anhui
JH-03	Gongju	20200608	Purchased online	Huangshan, Anhui
JH-04	Gongju	20200825	Purchased online	Huangshan, Anhui
JH-05	Gongju	20200908	Purchased online	Huangshan, Anhui
JH-06	Gongju	20200404	Purchased online	Huangshan, Anhui
JH-07	Hang-baiju	200407	Suzhou Tianling Traditional Chinese Medicine Tablet Co., Ltd.	Zhejiang
JH-08	Hang-baiju	191219	Suzhou Tianling Traditional Chinese Medicine Tablet Co., Ltd.	Zhejiang
JH-09	Hang-baiju	191209	Suzhou Li-liangji Traditional Chinese Medicine Co., Ltd.	Zhejiang
JH-10	Hang-baiju	200815	Bulk packaging in Xuzhou Deren Clinic	Zhejiang
JH-11	Hang-baiju	200404	Suzhou Lei-yunshang Traditional Chinese Medicine Co., Ltd.	Zhejiang
JH-12	Hang-baiju	191102	Suzhou Lei-yunshang Traditional Chinese Medicine Co., Ltd.	Zhejiang
JH-13	Taiju	200107	Purchased online	Zhejiang
JH-14	Taiju	191201	Purchased online	Zhejiang
JH-15	Taiju	200816	Suzhou Tianling Traditional Chinese Medicine Tablet Co., Ltd.	Zhejiang
JH-16	Taiju	20200830	Bulk packaging in Xuzhou Deren Clinic	Zhejiang
JH-17	Taiju	200506	Suzhou Tianling Traditional Chinese Medicine Tablet Co., Ltd.	Zhejiang
JH-18	Taiju	20200401	Bulk packaging in Xuzhou Deren Clinic	Zhejiang
JH-19	Boju	20200809	Purchased online	Bozhou, Anhui
JH-20	Boju	20200901	Purchased online	Bozhou, Anhui
JH-21	Boju	20200701	Purchased online	Bozhou, Anhui
JH-22	Boju	20200716	Purchased online	Bozhou, Anhui
JH-23	Boju	20200911	Bozhou Fumei Biotech Co., Ltd.	Bozhou, Anhui
JH-24	Boju	20200908	Purchased online	Bozhou, Anhui
JH-25	Huaiju	20200905	Bulk packaging in Cai-zhizhai Chinese medicine store	Jiaozuo, Henan
JH-26	Huaiju	20191220	Xinyuan Huaiyao Medicine Co., Ltd.	Jiaozuo, Henan
JH-27	Huaiju	20200908	Purchased online	Jiaozuo, Henan
JH-28	Huaiju	20200901	Purchased online	Jiaozuo, Henan
JH-29	Huaiju	20200909	Purchased online	Wenxian, Henan
JH-30	Huaiju	20200919	Purchased online	Jiaozuo, Henan

### Preparation of Mixed Standard and Sample Solution

The standard substances of ChA, 3,5-DCQA, L-7G, 4,5-DCQA, A-7G and K-3R were weighed accurately and dissolved in methanol to obtain the mixed standards solution containing the standards at the concentrations of 0.212, 0.248, 0.628, 0.420, 0.217, 0.742 mg ml^−1^, respectively. These standards solutions were sealed away from light and stored in −20°C refrigerator before use.

CM sample was crushed into powder. A total of 50 mg precisely-weighed powder was put into a 1.5 ml centrifuge tube, added 1 ml of 50% ethanol, and then sealed for extraction with ultrasonic treatment (500 W, 28 kHZ) for 30 min. After cooling, the solution was centrifuged at 12,000 rpm for 5 min at 4°C. A total of 0.5 ml supernatant was employed, diluted with 0.5 ml of methanol, and then filtered through 0.22 μm microporous membrane for use.

### Chromatographic Conditions

Chromatographic analysis was performed on an Agilent 1260 Infinity HPLC system consisted of G4212B 1260 DAD, G1322A 1260 Degasser, G1312B 1260 Bin Pump, G4226A 1290 Sampler, and G1316C 1290 TCC column temperature chamber. Samples were separated on an Agilent ZORBAX SB-C18 (4.6 × 250 mm, 5 μm) column with methanol (A) and 0.1% formic acid water solution (B) as the mobile phase, and the gradient elution was performed as follows : 85%–65%B, 5 min; 65%–55%B, 10 min; 55%–45%B, 20 min; 45%–35%B, 5 min; 35%–15%B, 5 min; and then 15%–85%B for 10 min to clean up the residues on the column. The flow rate was kept at 0.8 ml min^−1^ with detection wavelength of 348 nm, column temperature of 35°C, and injection volume of 15 μL.

### Antioxidant Activity Assay

DPPH clearance capacity was determined referring to the previous method with a slight modification (Zhang X. et al., 2021). Briefly, appropriate concentration of sample was mixed with 1 ml of 0.2 mM DPPH solution. After the reaction was carried for 30 min, the absorbance of the solution was determined at the wavelength of 517 nm. OH clearance capacity was determined as the published method with a minor modification (Jia et al., 2020). In brief, proper concentration of sample solution was added 1 ml of 6 mM FeSO_4_ and 1 ml of 8.8 mM H_2_O_2_. After being mixed and left for 10 min, 1 ml of 6 mM salicylic acid solution (dissolved in anhydrous ethanol) was added into it and mixed well, and then left it for another 30 min. After centrifuging at 12,000 rpm for 5 min, the absorbance of supernatant was measured at 510 nm. ABTS clearance capacity was measured according to the previous method with a slight modification (Gong et al., 2019). Briefly, suitable concentration of sample solution was added in 3.9 ml of ABTS working solution (1.76 ml of 140 mM potassium persulfate solution and 100 ml of 7 mM ABTS solution). After vortexing evenly and leaving at room temperature for 6 min, the absorbance of solution was recorded at 734 nm.

The blank control solution without free radical working solution and the negative control solution without antioxidants were prepared and determined as the same manner. The free radical scavenging capacity was calculated by Eq. 1.

$$\text{Free}\;\text{radical}\;\text{scavenging}\;\text{capacity}\;\left(\%\right)=\left(1-\frac{A_{a}-A_{b}}{A_{0}}\right)\times 100\%$$ (1)

Where *A*_*a*_ is the absorbance of sample solution which is a mixture of free radical working solution and sample solutions; *A*_*b*_ is the absorbance of blank control solution without free radical work solution; *A*_*0*_ is the absorbance of negative control solution without antioxidants.

### Statistical Analysis

SA was performed by the professional software “Similarity Evaluation System for Chromatographic Fingerprint of Traditional Chinese Medicine (Version 2012),” which was recommended by National Medical Products Administration of China. Statistical analysis was carried out with SPSS 23.0 (IBM, Armonk, NY, United States) and data were expressed as means ± standard deviation of at least three independent experimental results. Comparison among multiple groups was performed by one-way ANOVA, while differences between two groups were analyzed by using two-tailed Student’s *t*-test. The results with *p*-values of less than 0.05 were believed to be statistically significant.

## Results

### Chromatographic Fingerprint Analysis

Sample (JH-07) was selected to validate the methodology of chromatographic fingerprint analysis. The precision was evaluated by the relative standard deviation (RSD) of relative retention time (RRT) and relative peak area (RPA) of characteristic peaks from six consecutive times analysis of sample. The values of RRT and RPA of characteristic peaks were calculated with 3,5-DCQA (peak 2) as the reference peak, and the results showed that the RSD values of RRT and RPA of characteristic peaks were all less than 0.05% or 0.1%, respectively, which indicated that this analytical method possessed a good precision. The stability of sample solution at room temperature was investigated by measuring the variation of RRT and RPA of characteristic peaks at 0, 2, 6, 12, 24, and 48 h. The results showed that the RSD values of RRT and RPA were all less than 0.2% or 2.9%, respectively, which indicated that the sample solution was stable within 48 h. Six sample solutions were prepared in parallel and used to evaluate the reproducibility. The results showed that the RSD values of RRT and RPA of characteristic peaks were not more than 0.5% or 3.7%, respectively, which indicated that the method was reproducible.

**FIGURE 1:**
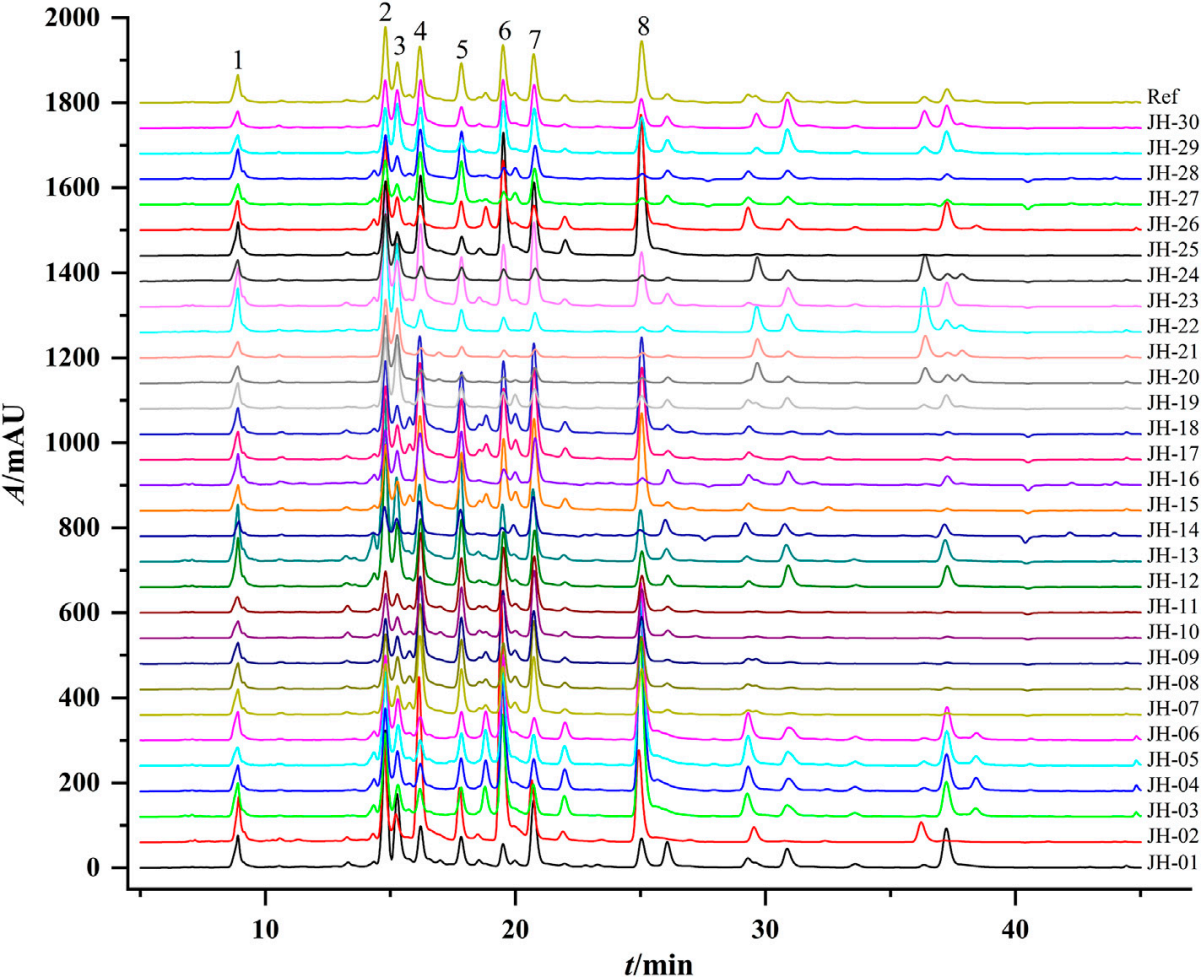
Representative HPLC chromatograms of 30 flower head samples of CMs. CM represents *Chrysanthemum morifolium;* R means the reference average fingerprint; JH-01∼JH30 mean 30 flower head samples of different accessions of CMs and their detailed information were listed in Table 1.

Thirty flower head samples of CMs were analyzed and their fingerprints were generated by using the average method with a time width of 0.1 (Figure 1). A total of eight peaks were identified as common peaks, and six characteristic peaks were identified as ChA, 3,5-DCQA, L-7G, 4,5-DCQA, A-7G and K-3R by comparing with the reference standards, respectively. By automatically matching the chromatographic peaks of 30 flower head samples of CMs to generate the average fingerprint, the similarities of different CMs were calculated by comparing them with the average fingerprint. The RPAs of eight common peaks of 30 flower head samples of CMs were calculated by the ratio of their peak areas with respect to the reference peak area (peak 2, 3,5-DCQA). From the Table 2 and Supplementary Table S1, we could find that the similarities of most CMs were more than 0.8 except JH-21 and JH-22 with a similarity value of 0.64 or 0.665, but the variation (RSD% value) of RPA of common peaks ranged from 27.2% to 86.3%.

**TABLE 2:** Similarity evaluation of different flower head samples of chrysanthemum samples.

No.	Accessions	Similarity	No.	Accessions	Similarity	No.	Accessions	Similarity
JH-01	Gongju	0.835	JH-11	Hang-baiju	0.903	JH-21	Boju	0.640
JH-02	Gongju	0.840	JH-12	Hang-baiju	0.917	JH-22	Boju	0.665
JH-03	Gongju	0.808	JH-13	Taiju	0.921	JH-23	Boju	0.956
JH-04	Gongju	0.827	JH-14	Taiju	0.807	JH-24	Boju	0.679
JH-05	Gongju	0.848	JH-15	Taiju	0.941	JH-25	Huaiju	0.909
JH-06	Gongju	0.831	JH-16	Taiju	0.845	JH-26	Huaiju	0.897
JH-07	Hang-baiju	0.931	JH-17	Taiju	0.952	JH-27	Huaiju	0.839
JH-08	Hang-baiju	0.932	JH-18	Taiju	0.946	JH-28	Huaiju	0.823
JH-09	Hang-baiju	0.923	JH-19	Boju	0.811	JH-29	Huaiju	0.923
JH-10	Hang-baiju	0.931	JH-20	Boju	0.681	JH-30	Huaiju	0.911

**FIGURE 2:**
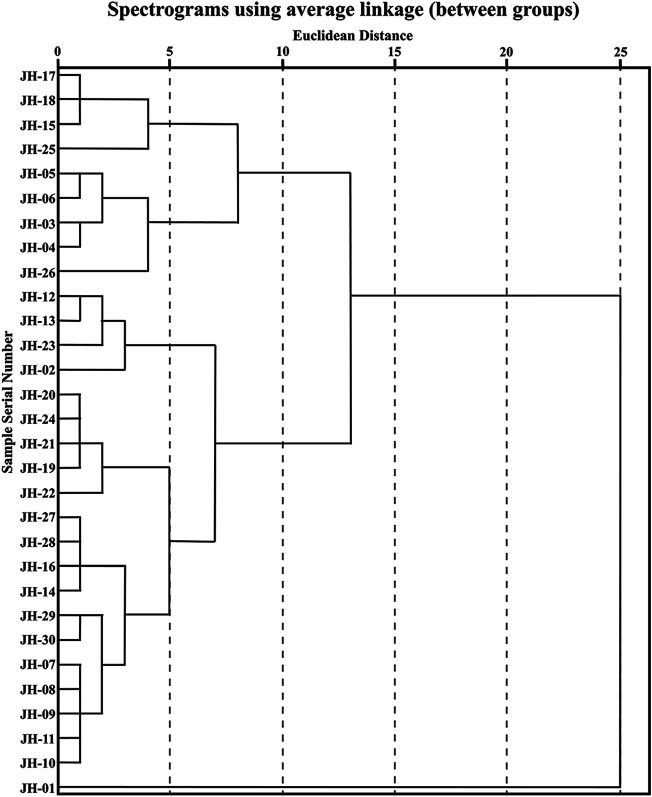
Phylogenetic cluster analysis of 30 flower head samples of CMs.

CA is a multivariate statistical technique for classifying sample or index components and is one of the most widely used multivariate statistical methods (Zhang Y. et al., 2021). The interval of intergroup connection and square Euclidean distance as the metric was used to establish a dendrogram of CA of 30 flower head samples of CMs, which is shown in Figure 2. The CA results of common peaks can basically classify different accessions of CMs into five classes at Euclidean distance of five and three classes at Euclidean distance of 10, and JH-01 was obviously far away from the others.

PCA is a multivariate statistical method widely used in multiple disciplines, which can be used to simplify data and quickly achieve visual identification of data (Cao et al., 2018). Factor analysis was performed on the eight common peaks of 30 flower head samples of CMs, and the component with an eigenvalue of more than 1 was taken as the extracted principal component (PC) according to the principle of principal component extraction (Liu et al., 2017). As shown in Table 3, the cumulative contribution of the first three PCs could reach 85.47%, indicating that the extracted three PCs could basically respond to the main information of the samples. The eigenvalue in PC1 reached 3.04 with the variance contribution rate of 38.04%, and the peaks with higher loadings were mainly ChA, L-7G, 4,5-DCQA, A-7G, and K-3R, indicating that these five peaks mainly reflected the information of PC1. The eigenvalue of PC2 was 2.284 with the variance contribution rate of 28.55%, and the peaks with higher loadings were ChA, 3,5-DCQA, and Peak 3 (unknown peak 1), indicating that these three peaks mainly reflected the information of PC2. The eigenvalue of PC3 was 1.511 with the variance contribution rate of 18.88%, and the peaks with higher loadings were A-7G and Peak 8 (unknown peak 2), indicating that the two peaks mainly reflected the information of PC3.

**TABLE 3:** Principal component analysis and factor loading matrix of 30 flower head samples of CMs.

	Principal components							
	1	2	3	4	5	6	7	8
Initial eigenvalue	3.04	2.28	1.51	0.56	0.40	0.12	0.06	0.03
Variance contribution rate (%)	38.04	28.55	18.88	6.99	4.96	1.52	0.70	0.35
Cumulative contribution rate (%)	38.04	66.60	85.47	92.47	97.43	98.95	99.65	100
**Characteristic peak**	**Principal component factor loading matrix**							
Peak 1 (ChA)	0.63	0.66	0.14	—	—	—	—	—
Peak 2 (3,5-DCQA)	0.45	0.85	0.11	—	—	—	—	—
Peak 3 (Unknown 1)	−0.14	0.93	−0.11	—	—	—	—	—
Peak 4 (L-7G)	0.82	−0.35	−0.30	—	—	—	—	—
Peak 5 (4,5-DCQA)	0.76	0.02	−0.41	—	—	—	—	—
Peak 6 (A-7G)	0.68	−0.22	0.61	—	—	—	—	—
Peak 7 (K-3R)	0.76	−0.24	−0.43	—	—	—	—	—
Peak 8 (Unknown 2)	0.38	−0.18	0.81	—	—	—	—	—

**FIGURE 3:**
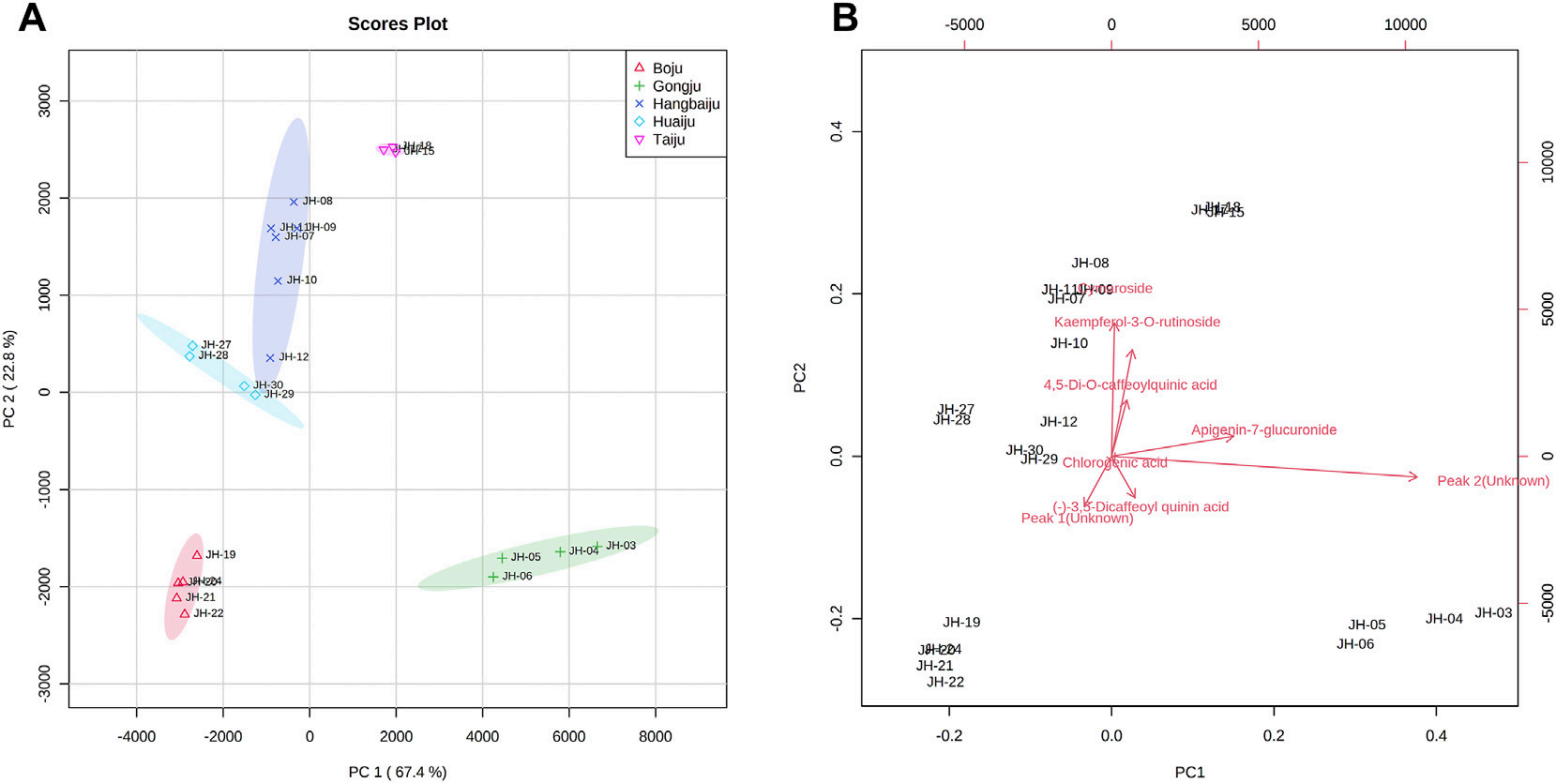
Two-dimensional score plots **(A)** and loading plots **(B)** of PCA of different accessions of CMs.

To further distinguish the effects of PCs, the data were imported into MetaboAnalyst 4.0 (http://www.metaboanalyst.ca) to obtain two-dimensional analysis plot and loadings plot using eight common peaks as variables and original grouping as a reference. According to the results of CA, some samples with excessive deviations were removed. The results in Figure 3A showed that the contribution values of PC1 and PC2 reached 67.4% and 22.8%, respectively, and the various accessions of CMs were well separated. As shown in Figure 3B, L-7G, K-3R, 4,5-DCQA, unknown peak 1, and 3,5-DCQA were the main components to distinguish the difference between Taiju, Huaiju, Hang-baiju, and Boju, while unknown peak 2 and A-7G were the main components to distinguish the difference between other kinds of CMs and Gongju.

### Quantification of the Eight Common Peak Components in CMs

**FIGURE 4:**
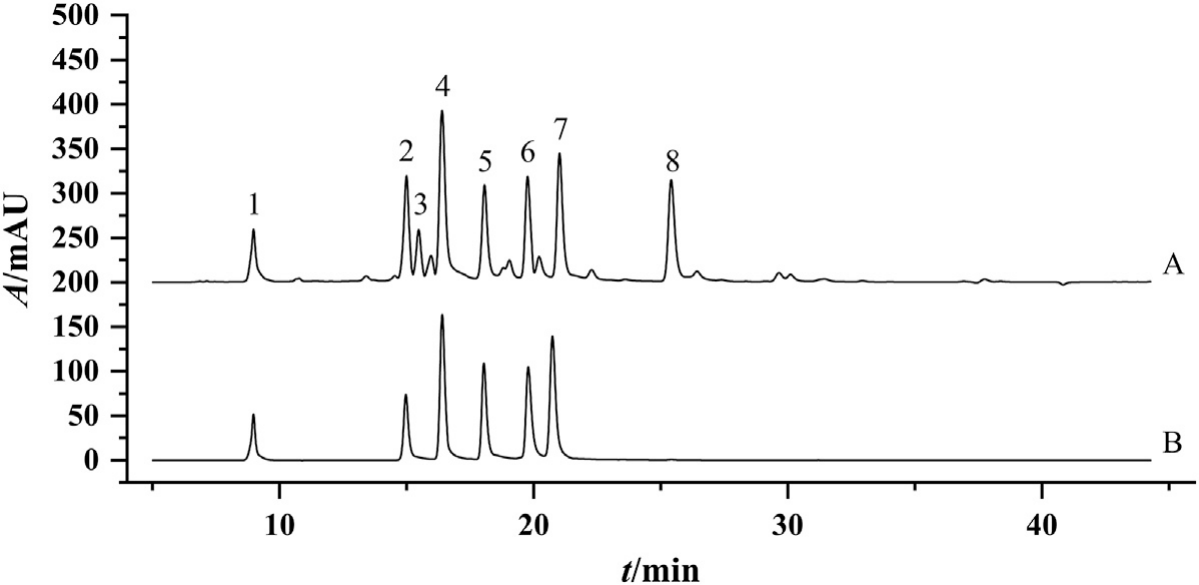
Representative chromatogram of the Sample JH-07 **(A)** and mixed standard solution **(B)**. 1: ChA; Peak 2, 3,5-DCQA; Peak 3, Unknown peak 1; Peak 4, L-7G; Peak 5, 4.5-DCQA; Peak 6, A-7G; Peak 7, K-3R; Peak 8, Unknown peak 2.

To accurately find the difference among 30 flower head samples of CMs, the common peaks were further quantified. Chromatographic separation of 30 flower head samples of CMs was performed under the set condition of this study, and the representative chromatograms of the reference standards and sample were shown in Figure 4. The resolutions of the eight common peaks were all more than 1.5, and their theoretical numbers of plate were all better than 8,000.

#### Determination of Relative Correction Factors

As two unknown common peaks (Peak 3 and Peak 8) could not be identified by comparing with chemical data and the reference standards of CM, thus, their quantifications were performed by the relative correction factor method referring to the previous studies (Hou et al., 2011; Zhao et al., 2021). This method assumed that if the structure of an unknown peak (without standard) was similar with a known peak (with the standard), the quantification of the unknown peak could be performed by the content of the known peak multiplying by the relative correction factor.

In this study, to find the relative correction factors of two unknown peaks, different concentrations of the same sample solution were determined, and the relative correction factor (*f*_*s*_) was determined by calculating Eq. 2, and the *f*_*s*_ with a constant value and lower variation (RSD%) was selected to quantify the unknown peak.

$$\mathrm{Relative}\;\mathrm{correction}\;\mathrm{factor}\;f_{s}=\frac{A_{s}}{A_{i}}=\frac{C_{s}}{C_{i}}$$ (2)

where *A*_*s*_ and *C*_*s*_ represent the peak area and concentration of known compound including ChA, 3,5-DCQA, L-7G, 4,5-DCQA, A-7G, or K-3R, respectively. *A*_*i*_ and *C*_*i*_ mean the peak area and concentration of unknown compound (Peak 3 and Peak 8), respectively.

As shown in Supplementary Tables S2, S3, the RSD value of *f*_*s*_ of unknown peak 1 to L-7G was 1.95%, while the RSD value of *f*_*s*_ of unknown peak 2 to K-3R was 3.32%, which were much lower than that of other known compounds. Thus, the linear equation of L-7G and K-3R were used to calculate the concentration of unknown peak 1 or unknown peak 2 with a *f*_*s*_ value of 0.93 or 1.37, respectively.

#### Quantitative Method Validation

A total of five series of standard solutions of six compounds including ChA, 3,5-DCQA, L-7G, 4,5-DCQA, A-7G, and K-3R were used to determine the linear range by an external standard method. Calibration curves were generated by plotting the peak areas (*y*) versus the corresponding concentrations (*x*, μg mL^−1^). A series of diluted solutions of six reference standards were used to determine limit of detection (LOD) and limit of quantification (LOQ), which were defined as the signal-to-noise (S/N) ratios of 3 and 10, respectively. As shown in Table 4, the correlation coefficients were all better than 0.9951 for all analytes and the range of LODs and LOQs for all compounds were in the ranges of 0.05–0.14 μg ml^−1^, and 0.12–0.51 μg ml^−1^, respectively, which indicated that this method possessed a good linearity and sensitivity.

**TABLE 4:** Linear relationship of standard substances from components of CMs.

Components	Regression equation^a^	*R* ^*2*^	Linear range (μg ml^−1^)	LOD^b^ (μg ml^−1^)	LOQ^c^ (μg ml^−1^)
ChA	*y* = 8.6378*x*–20.26	0.9989	16.4–81.8	0.05	0.16
3,5-DCQA	*y* = 12.211*x*–111.77	0.9984	19.1–95.7	0.06	0.19
L-7G	*y* = 10.674*x*–0.386	0.9985	48.5–242.4	0.14	0.49
4,5-DCQA	*y* = 11.367*x*–293.87	0.9895	32.4–162.1	0.09	0.32
A-7G	*y* = 22.281*x*–297.62	0.9951	16.8–83.8	0.05	0.17
K-3R	*y* = 7.0582*x*–19.461	0.9991	27.3–286.4	0.08	0.27

a *y* and *x* are the peak areas and concentrations (μg·mL^−1^) of the analytes, respectively.

b The limit of detection (LOD) was defined as the concentration for which the signal-to-noise ratio was 3.

c the limit of quantification (LOQ) was defined as the concentration for which the signal-to-noise ratio was 10.

The precision of the proposed method was determined by injecting mixed standard solution for six consecutive times, and the RSD values of the peak areas of ChA, 3,5-DCQA, L-7G, 4,5-DCQA, A-7G, and K-3R were used to evaluate the precision. The results showed that the RSD values of six reference standards were 0.26%, 0.38%, 0.30%, 0.34%, 0.26%, and 0.27%, respectively. The repeatability was investigated with six independently prepared sample solutions of JH-07, one of which was injected into the apparatus at 0, 2, 4, 8, 12, and 24 h, separately, to determine the stability of the solution. The results showed that the RSD values of six analytes in the six independently prepared sample solutions were 1.84%, 0.64%, 0.97%, 1.48%, 1.06%, and 1.80%, respectively, and the RSD values of six analytes in one sample solution within 24 h were 0.65%, 0.73%, 1.81%, 0.88%, 1.82%, and 1.20%, respectively. These results hinted that this method possessed a good precision, repeatability, and stability.

The accuracy was confirmed by the recovery measured by standard addition method. Six different amounts of the reference standards were added to a real sample for which the concentrations of the compounds of interest were known. The mixtures were treated and analyzed using the method in this study. The accuracy was expressed as the percentage of the analytes recovered by the assay. As shown in Supplementary Table S4, the recoveries of the components ranged from 91.6% to 110.2% with the RSD values of 2.4%–6.2%, which indicated that the method enabled highly accurate simultaneous analysis of the six analytes.

#### Samples Determination

**FIGURE 5:**
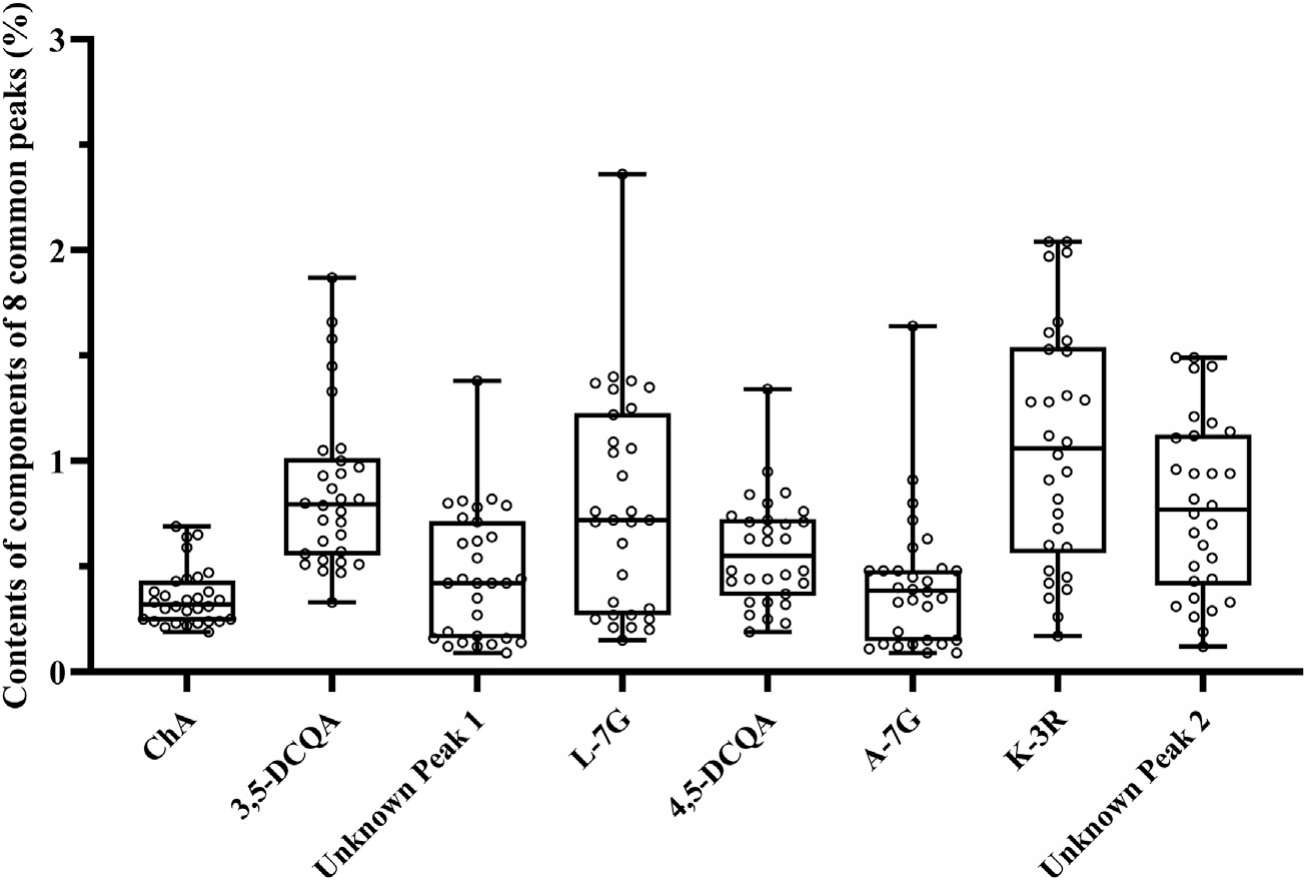
The box diagram of the contents of eight common peaks in 30 flower head samples of CMs.

The validated method was used to determine the contents of eight common peaks in 30 flower head samples of CMs, among which 2 unknown peaks were quantified using the relative correction factor method, while six known peaks were assayed by the external standard method. As shown in Table 5, the variation (RSD) of the contents of eight common peaks of 30 flower head samples of different accessions of CMs ranged from 38.7% to 77.3%. Even for the same accessions of CM, the variation (RSD) of the contents of eight common peaks were still enormous, such as Gongju, Hang-baiju, Taiju, Boju, and Huaiju with the RSD value ranges of 21.4%–123.0%, 11.7%–64.7%, 31.3%–60.9%, 32.7%–119.8%, and 38.7%–77.3%, respectively. From the Figure 5, we could vividly observe the scattered distribution of the contents of eight common peaks in 30 flower head samples of CMs.

**TABLE 5:** Contents of eight common peaks in 30 batches of CMs (%, g/g).

No.	Accessions	ChA	3,5-DCQA	Unknown peak 1	L-7G	4,5-DCQA	A-7G	K-3R	Unknown peak 2
JH-01	Gongju	0.65	1.00	1.38	2.36	0.72	1.64	1.28	0.94
JH-02		0.43	1.58	0.35	0.61	0.44	0.19	1.57	1.14
JH-03		0.44	0.93	0.16	0.27	0.46	0.80	0.68	0.50
JH-04		0.34	0.97	0.19	0.33	0.48	0.72	0.60	0.44
JH-05		0.24	1.06	0.16	0.27	0.48	0.63	0.59	0.43
JH-06		0.38	0.94	0.14	0.25	0.44	0.59	0.42	0.31
RSD^a^		33.1	23.1	123.0	122.2	21.4	62.9	53.4	53.0
JH-07	Hangbaiju	0.30	0.57	0.71	1.22	0.62	0.34	1.29	0.94
JH-08		0.31	0.62	0.78	1.34	0.67	0.33	1.52	1.11
JH-09		0.25	0.48	0.73	1.25	0.63	0.49	1.12	0.82
JH-10		0.21	0.51	0.44	0.76	0.70	0.31	1.53	1.12
JH-11		0.19	0.47	0.62	1.06	0.74	0.45	1.28	0.94
JH-12		0.69	1.66	0.54	0.93	0.95	0.39	1.31	0.96
RSD^a^		56.9	64.7	20.2	20.1	17.0	18.7	11.7	11.7
JH-13	Taiju	0.64	1.87	0.61	1.04	1.34	0.40	1.61	1.18
JH-14		0.22	0.33	0.27	0.46	0.42	0.11	0.91	0.66
JH-15		0.30	0.72	0.79	1.35	0.80	0.48	2.04	1.49
JH-16		0.36	0.65	0.42	0.72	0.76	0.15	1.09	0.79
JH-17		0.33	0.80	0.81	1.38	0.84	0.48	2.04	1.49
JH-18		0.31	0.79	0.82	1.40	0.85	0.48	1.99	1.45
RSD^a^		40.3	60.9	37.3	37.3	35.3	49.6	31.3	31.5
JH-19	Boju	0.34	0.76	0.13	0.21	0.33	0.15	0.45	0.33
JH-20		0.23	0.82	0.12	0.20	0.19	0.09	0.35	0.26
JH-21		0.23	0.71	0.09	0.15	0.23	0.09	0.17	0.12
JH-22		0.59	1.33	0.14	0.25	0.37	0.13	0.39	0.29
JH-23		0.45	1.45	0.80	1.37	0.71	0.43	1.97	1.44
JH-24		0.29	0.82	0.12	0.21	0.27	0.13	0.26	0.19
RSD^a^		39.8	32.7	119.2	119.8	53.7	76.2	113.5	113.2
JH-25	Huaiju	0.47	0.87	0.64	1.09	0.32	0.91	1.66	1.21
JH-26		0.38	1.05	0.17	0.30	0.43	0.48	0.48	0.35
JH-27		0.24	0.53	0.44	0.76	0.63	0.13	0.82	0.60
JH-28		0.35	0.51	0.42	0.71	0.71	0.12	0.75	0.54
JH-29		0.25	0.52	0.42	0.71	0.25	0.38	1.03	0.75
JH-30		0.24	0.56	0.42	0.72	0.33	0.35	0.95	0.70
RSD^a^		29.4	34.1	35.7	35.1	41.6	73.4	41.9	41.9
RSD^b^		38.7	44.4	66.5	66.3	44.6	77.3	53.3	53.2

RSD^a^, represents the variation of the contents of eight common peaks from the same accessions of CM; RSD^b^, represents the variation of the contents of eight common peaks from 30 flower head samples of different accessions of CM.

### Prediction of Q-Marker of CMs Based on Antioxidant Activity

To find the Q-marker, the *in-vitro* chemical antioxidant activity of CMs was first determined, and then the correlation analysis between the chemical antioxidant activity and the contents of eight common peaks was performed. To minimize the deviation of Q-marker as much as possible, some CMs samples that were excessively deviated from most samples according to the results of chemometrics were removed. Finally, three samples of each accession of CMs and a total of 15 CMs were selected to be evaluated the chemical antioxidant activities. As shown in Table 6, the DPPH, OH, and ABTS clearance rate of different CMs ranged from 28.34% to 85.35%, 55.72% to 81.18%, and 49.65%–93.75%, respectively.

**TABLE 6:** Free radical scavenging rate of different species of CMs (*n* = 3).

No.	Accessions	Free radical clearance rate (%)			No.	Accessions	Free radical clearance rate (%)		
		DPPH·	OH·	ABTS·			DPPH·	OH·	ABTS·
JH-02	Gongju	73.41 ± 0.40	79.25 ± 0.14	90.22 ± 0.48	JH-17	Taiju	50.37 ± 0.20	71.50 ± 0.07	79.20 ± 0.90
JH-04	Gongju	43.33 ± 0.23	71.14 ± 0.19	70.20 ± 0.90	JH-19	Boju	33.29 ± 0.38	72.37 ± 0.19	64.97 ± 0.74
JH-06	Gongju	37.92 ± 0.14	71.26 ± 0.14	69.21 ± 0.96	JH-23	Boju	58.88 ± 0.49	72.53 ± 0.07	90.01 ± 0.48
JH-09	Hang-baiju	42.93 ± 0.10	67.61 ± 0.26	70.52 ± 0.90	JH-24	Boju	28.51 ± 0.06	75.11 ± 0.07	67.13 ± 0.69
JH-10	Hang-baiju	43.44 ± 0.10	55.72 ± 0.25	72.53 ± 1.01	JH-28	Huaiju	38.59 ± 0.26	71.63 ± 0.07	66.42 ± 0.95
JH-12	Hang-baiju	70.77 ± 0.45	77.86 ± 0.12	88.21 ± 0.58	JH-29	Huaiju	32.51 ± 0.17	56.01 ± 0.07	59.92 ± 0.80
JH-13	Taiju	85.35 ± 0.16	81.18 ± 0.25	93.75 ± 0.11	JH-30	Huaiju	35.28 ± 0.79	72.28 ± 0.07	61.37 ± 0.85
JH-14	Taiju	28.34 ± 0.06	63.76 ± 0.38	49.65 ± 0.80					

The concentrations of sample solutions for the determination of DPPH, OH, or ABTS· clearance rate were 1.0, 10.0 or 5.0 mg/ml, respectively.

Pearson correlation analysis between the chemical antioxidant activities and the contents of eight common peaks was then further performed, and the results (Table 7) showed the contents of ChA, 3,5-DCQA, and unknown peak 1 were significantly correlated with ABTS·, DPPH·, and OH clearance capacities, while the contents of 4,5-DCQA and K-3R were significantly correlated with ABTS and DPPH clearance capacities. The correlation coefficients of the above five compounds with the antioxidant activity ranked as follows: 3,5-DCQA ˃ ChA ˃ unknown peak 1 ˃ 4,5-DCQA ˃ K-3R. Thus, the above five compounds were selected as the Q-markers of chemical antioxidant activity of CMs.

**TABLE 7:** Correlation analysis between the antioxidant activity and the contents of eight common peaks from the different CMs.

Characteristic peak	ABTS clearance capacity	DPPH clearance capacity	OH clearance capacity
ChA	0.778^**^ (0.001)	0.799^**^ (0.000)	0.709^**^ (0.003)
3,5-DCQA	0.899^**^ (0.000)	0.900^**^ (0.000)	0.771^**^ (0.001)
Unknown peak 1	0.582^*^ (0.023)	0.628^*^ (0.012)	0.650^**^ (0.009)
L-7G	0.512 (0.051)	0.462 (0.083)	0.039 (0.890)
4,5-DCQA	0.713^**^ (0.003)	0.800^**^ (0.000)	0.377 (0.166)
A-7G	0.162 (0.565)	0.056 (0.843)	−0.008 (0.979)
K-3R	0.622^*^ (0.013)	0.602^*^ (0.018)	0.056 (0.844)
Unknown peak 2	0.073 (0.795)	−0.023 (0.986)	0.021 (0.942)

The data means the correlation coefficient (*p* value).

### Quality Evaluation of Commercially Available CMs Using the Q-Marker of Antioxidant Activity

The contents of ChA, 3,5-DCQA, unknown peak 1, 4,5-DCQA, and K-3R in 30 flower head samples of different accessions of commercially available CMs were further determined, and the results were shown in Table 8. To evaluate the quality of CMs in a relatively objective way, this study set up four grades of excellent, good, medium, and poor according to the contents of five compounds. As the content changes of five compounds were inconsistent among different accessions of CMs, we further set up five different sub-grades according to the contents of five different compounds. The top 10%, ranking of 11%–30%, 31%–90%, and 91%–100% of the contents of ChA, 3,5-DCQA, unknown peak 1, 4,5-DCQA, or K-3R were respectively set as the excellent, good, medium, and poor, and assigned the value of “1”; “2,” “3,” and “4,” respectively. As the contribution degree of five compounds to the chemical antioxidant activity of CMs were different, the proportions of 3,5-DCQA, ChA, unknown peak 1, 4,5-DCQA, and K-3R in the calculation of final grade were set as 30%, 25%, 20%, 15%, and 10% referring to their corresponding rank, respectively. Thus, the final grades of different accessions of CMs were calculated by the following Equation: Grade = Sub-grade (3,5-DCQA) × 0.3 + Sub-grade (ChA) × 0.25 + Sub-grade (unknown peak 1) × 0.2 + Sub-grade (4,5-DCQA) × 0.15 + Sub-grade (K-3R) × 0.1, and shown in Table 8. From Table 8, we could find that 30 flower head samples of different accessions of CMs were sorted as different grades. Two of them (JH-12 and JH-13), respectively from Hang-baiju and Taiju, were assigned as the excellent; 11 of them were evaluated as the good; and two of them (JH-14 and JH-21), respectively from Taiju and Boju, were sorted as the poor. These results further hinted that there existed wide quality variation between different accessions of CMs, and even for the same accession of CMs.

**TABLE 8:** Evaluation of commercially available CMs by Q-marker based on antioxidant activity.

No.	Accessions	Contents (%, g/g)					Grade
		ChA	3,5-DCQA	Unknown peak 1	4,5-DCQA	K-3R	
JH-01	Gongju	0.65	1.00	1.38	0.72	1.28	Good
JH-02		0.43	2.20	0.35	0.44	1.57	Good
JH-03		0.44	1.27	0.16	0.46	0.68	Good
JH-04		0.34	1.32	0.19	0.48	0.60	Medium
JH-05		0.24	1.46	0.16	0.48	0.59	Medium
JH-06		0.38	1.29	0.14	0.44	0.42	Good
JH-07	Hang-baiju	0.30	0.76	0.71	0.62	1.29	Medium
JH-08		0.31	0.84	0.78	0.67	1.52	Medium
JH-09		0.25	0.64	0.73	0.63	1.12	Medium
JH-10		0.21	0.68	0.44	0.70	1.53	Medium
JH-11		0.19	0.62	0.62	0.74	1.28	Medium
JH-12		0.69	2.31	0.54	0.95	1.31	Excellent
JH-13	Taiju	0.64	2.60	0.61	1.34	1.61	Excellent
JH-14		0.22	0.43	0.27	0.42	0.91	Poor
JH-15		0.30	0.98	0.79	0.80	2.04	Good
JH-16		0.36	0.87	0.42	0.76	1.09	Medium
JH-17		0.33	1.09	0.81	0.84	2.04	Good
JH-18		0.31	1.08	0.82	0.85	1.99	Good
JH-19	Boju	0.34	1.03	0.13	0.33	0.45	Medium
JH-20		0.23	1.12	0.12	0.19	0.35	Medium
JH-21		0.23	0.96	0.09	0.23	0.17	Poor
JH-22		0.59	1.84	0.14	0.37	0.39	Good
JH-23		0.45	2.00	0.80	0.71	1.97	Good
JH-24		0.29	1.12	0.12	0.27	0.26	Medium
JH-25	Huaiju	0.47	1.19	0.64	0.32	1.66	Good
JH-26		0.38	1.44	0.17	0.43	0.48	Good
JH-27		0.24	0.70	0.44	0.63	0.82	Medium
JH-28		0.35	0.68	0.42	0.71	0.75	Medium
JH-29		0.25	0.69	0.42	0.25	1.03	Medium
JH-30		0.24	0.75	0.42	0.33	0.95	Medium

## Discussion

To obtain a chromatographic separation of CM, chromatographic conditions were first optimized. As CM is rich in flavonoids and phenolic acids, referring to our group’s previous experience and study (Tang et al., 2010), Agilent ZORBAX SB-C18 column was thus selected as the stationary phase, and four mobile phase systems including methanol-water, methanol-0.1% formic acid water solution, methanol-0.2% formic acid water solution, and 0.1% formic acid methanol solution-0.1% formic acid water solution were investigated, respectively. The results showed that lower resolution and fewer chromatographic peaks could be obtained when using methanol-water as the mobile phase, while all components could be fully separated using the other three kinds of mobile phase. Considering the environmental protection and low consumption, methanol-0.1% formic acid water was finally chosen as the mobile phase in this study. The influences of column temperature at 30°C, 35°C, 37°C, and 40°C on the chromatographic separation were respectively observed, and the results showed that the column temperature at 35°C could obtain the lower back pressure, higher response, and better baseline. After repeated test, the optimal grade elution mode listed in this study was finally obtained.

Flavonoids and phenolic acids are the active substances responsible for the antioxidant activity of CM (Chen et al., 2015; Peng et al., 2019; Sun et al., 2021), thus, the contents of total phenolic acids and total flavonoids were thus selected as the evaluation indexes of extraction methods. In this experiment, extraction method, solvent, time and the ratio of liquid to solid were optimized for the preparation of the sample solution, respectively. Three extraction methods including water decoction, reflux extraction, and ultrasonic method were separately investigated, and the results showed that the extraction rate of ultrasonic extraction method was significantly higher than the other two extraction methods (Supplementary Table S5). The extraction solvents including pure water, 30%, 50%, 70%, 100% methanol or ethanol were then observed, and it was found that 50% ethanol could obtain the best extraction rate for the total phenolic acids and total flavonoids (Supplementary Table S6). The ultrasonic extraction for 10, 15, 20, 30, and 40 min was further investigated, and the results showed that when the extraction time was more than 30 min, the extraction rate could not be obviously increased (Supplementary Table S7). Finally, the ration of liquid to solid was optimized, and it was found that when the ratio was more than 1:20, the extraction rate could not be significantly elevated (Supplementary Table S8).

In the quality control of TCM, chromatographic fingerprint analysis plays an important role, which can give an overall view of the characteristics of nearly all the components. However, it can only show results of similarity calculated on the basis of the relative value using a pre-selected marker compound as a reference and cannot reveal the possible content variation (Tang et al., 2010). Our results also support this fact. In our study, the similarities of most CMs were more than 0.8 except JH-21 and JH-22, however, the peak area variation (RSD value) of common peak ranged from 42.4% to 86.3%. Chemometrics can provide further data mining of chromatographic fingerprint. For example, CA can group the similar samples into several classes (Zhao et al., 2020) and visually explore the similarity of different accessions of CMs. Our results of CA revealed that the same accessions of CMs were not clustered into one class, which hinted that the difference of CMs was related to not only the species, but also the origin, harvesting season, maturity, and storage and processing condition (Chen X. et al., 2020; Khan et al., 2020). PCA is often used to explain differences between samples and to obtain information on the variables that primarily influence sample similarity and variability (Šamec et al., 2016). Our results of PCA indicated that the peak areas of the eight common peaks all contributed to the difference between different species of CMs.

The combination of chromatographic fingerprint and chemometrics can explore the similarity and the reason of the difference between different accessions of CMs. However, these analytical strategies are all based on the peak areas of common peaks. Thus, multiple ingredients quantification, especially the common peaks quantification, is considered as an important complementary to chromatographic fingerprint analysis for the quality control of TCM (Tang et al., 2010; Yang et al., 2011). Our results of accurate quantification of the common peaks further verified that the RSD values of their contents possessed significant variations whether for the different accessions of CMs or for the same accession of CMs. The results were consistent with the peak areas variation of the common peaks in the chromatographic fingerprint analysis, and further verified that the species, origin, harvesting season, maturity, and storage and processing conditions all contributed to the quality variation of CMs.

Although the quantification of the common peak can provide an important complementary to chromatographic fingerprint analysis for the quality control of TCM, however, different compounds in the common peaks possessed the different contribution to the biological activity of TCM. Thus, how to control the quality of TCM according to the contents of common peaks is still a challenge. Recently, Q-marker based on biological activity provides a new strategy for the quality control of TCM. As anti-oxidant activity is the basis of pharmacological effects of CM, the correlation analysis between the chemical anti-oxidant activity and the contents of common peaks was further performed to explore the Q-marker of CM in this study. Our results hinted that ChA, 3,5-DCQA, unknown peak 1, 4,5-DCQA, and K-3R might be used as the Q-markers of CMs, which was partly consistent with a previous study (Zhou et al., 2019). More importantly, we successfully utilized the Q-markers to evaluate the quality of 30 flower head samples of different accessions of commercially available CMs according to their contributions to the chemical anti-oxidant activity of CMs, and the results showed that the quality of JH-21 was consistent with the results of chromatographic fingerprint analysis. The synthesis and accumulation of phytochemical compositions in plant tissues are influenced by the genotype, growing environment, and their interaction (Kiani et al., 2021). Hang-baiju and Taiju, two famous-region drugs of Zhejiang Province of China, are generally considered as higher quality of CMs (Gong et al., 2019). Our results also showed that JH-12 and JH-13, respectively from Hang-baiju and Taiju, were assigned as the excellent based on the evaluation of Q-marker. However, JH-14, a flower head sample from Taiju, was sorted as the poor, which hinted that JH-14 might be an adulteration product. Those results also indicated that it was unreliable to evaluate the quality of CMs according to geographical origins.

Although this study preliminarily screened chemical anti-oxidant quality markers, there are also some limitations. Firstly, quality markers were screened only from eight components in this study, and some active components may be omitted. Therefore, the analysis of chemical components needs to be further expanded and screened. Secondly, the screening of anti-oxidant quality markers of CM was based on the DPPH, OH, and ABTS clearance rate. Those methods are simply chemical tests and cannot provide the definite evidence of anti-oxidant activity of CM. Thus, *in vivo* anti-oxidant activity using cell or animal as model needs to be further explored in future research. In addition, CM also has many kinds of activities include antiviral, anti-inflammatory, anti-bacterial, anti-tumor, anti-infective, and anti-inflammatory effects except anti-oxidant. Therefore, quality markers that can comprehensively mirror varies kinds of biological activities of CM need to be further studied.

This study identified the Q-markers of CM through the analysis of chromatographic fingerprint, quantification of common peaks, chemical anti-oxidant activity and the spectrum-effect relationship. The quality control of CM was implemented with the chromatographic fingerprint, multiple ingredients quantification and Q-marker determination. Overall, this study has discovered that the Q-marker based quality assessment of CM was feasible and practical. This study also serves as a reference for Q-marker selections and quality control of other TCMs.

## Data Availability

The original contributions presented in the study are included in the article/Supplementary Material, further inquiries can be directed to the corresponding author.
