# Supplementary material for: Chemical Antioxidant Quality Markers of Chrysanthemum morifolium Using a Spectrum-Effect Approach
Source: Front Pharmacol. 2022 Feb 7;13:809482. doi: 10.3389/fphar.2022.809482 (PMC8859431; doi:10.3389/fphar.2022.809482)
Supplement: Supplementary file 1 [file DataSheet1.docx]

Supplementary Material

**Table S1** The RPA of the common peaks of different flower head samples of CMs

| No. | Peak 1 | Peak 2 (S) | Peak 3 | Peak 4 | Peak 5 | Peak 6 | Peak 7 | Peak 8 |
| --- | --- | --- | --- | --- | --- | --- | --- | --- |
| JH-01 | 0.47 | 1.00 | 0.28 | 2.14 | 0.60 | 3.01 | 0.76 | 1.25 |
| JH-02 | 0.19 | 1.00 | 0.66 | 0.34 | 0.20 | 0.16 | 0.58 | 0.25 |
| JH-03 | 0.34 | 1.00 | 0.35 | 0.27 | 0.38 | 1.53 | 0.44 | 3.29 |
| JH-04 | 0.25 | 1.00 | 0.49 | 0.31 | 0.38 | 1.31 | 0.37 | 2.90 |
| JH-05 | 0.16 | 1.00 | 0.40 | 0.23 | 0.34 | 1.02 | 0.33 | 2.22 |
| JH-06 | 0.29 | 1.00 | 0.47 | 0.24 | 0.35 | 1.08 | 0.26 | 2.49 |
| JH-07 | 0.39 | 1.00 | 0.62 | 2.00 | 0.91 | 0.99 | 1.38 | 1.14 |
| JH-08 | 0.36 | 1.00 | 0.64 | 1.99 | 0.90 | 0.87 | 1.49 | 1.28 |
| JH-09 | 0.39 | 1.00 | 0.56 | 2.46 | 1.10 | 1.81 | 1.45 | 1.53 |
| JH-10 | 0.29 | 1.00 | 0.45 | 1.39 | 1.16 | 0.98 | 1.85 | 1.34 |
| JH-11 | 0.30 | 1.00 | 0.40 | 2.14 | 1.37 | 1.68 | 1.70 | 1.12 |
| JH-12 | 0.30 | 1.00 | 0.47 | 0.50 | 0.49 | 0.38 | 0.46 | 0.29 |
| JH-13 | 0.24 | 1.00 | 0.51 | 0.50 | 0.63 | 0.34 | 0.51 | 0.37 |
| JH-14 | 0.50 | 1.00 | 0.64 | 1.36 | 1.00 | 0.32 | 1.75 | 0.32 |
| JH-15 | 0.30 | 1.00 | 0.40 | 1.72 | 0.95 | 1.14 | 1.71 | 2.09 |
| JH-16 | 0.41 | 1.00 | 0.63 | 1.03 | 1.00 | 0.28 | 1.02 | 0.16 |
| JH-17 | 0.29 | 1.00 | 0.43 | 1.58 | 0.89 | 1.01 | 1.53 | 1.74 |
| JH-18 | 0.28 | 1.00 | 0.35 | 1.62 | 0.91 | 1.04 | 1.51 | 1.86 |
| JH-19 | 0.33 | 1.00 | 0.94 | 0.26 | 0.30 | 0.24 | 0.35 | 0.24 |
| JH-20 | 0.20 | 1.00 | 0.88 | 0.22 | 0.11 | 0.07 | 0.25 | 0.09 |
| JH-21 | 0.24 | 1.00 | 1.02 | 0.20 | 0.17 | 0.11 | 0.13 | 0.10 |
| JH-22 | 0.31 | 1.00 | 0.79 | 0.17 | 0.19 | 0.11 | 0.17 | 0.05 |
| JH-23 | 0.22 | 1.00 | 0.43 | 0.85 | 0.40 | 0.49 | 0.80 | 0.50 |
| JH-24 | 0.26 | 1.00 | 0.86 | 0.23 | 0.19 | 0.17 | 0.19 | 0.10 |
| JH-25 | 0.39 | 1.00 | 0.30 | 1.14 | 0.25 | 1.87 | 1.14 | 2.18 |
| JH-26 | 0.26 | 1.00 | 0.37 | 0.26 | 0.30 | 0.77 | 0.27 | 1.50 |
| JH-27 | 0.34 | 1.00 | 0.41 | 1.35 | 1.00 | 0.28 | 0.95 | 0.19 |
| JH-28 | 0.52 | 1.00 | 0.49 | 1.32 | 1.20 | 0.26 | 0.90 | 0.16 |
| JH-29 | 0.35 | 1.00 | 1.31 | 1.29 | 0.28 | 1.23 | 1.22 | 0.98 |
| JH-30 | 0.31 | 1.00 | 0.94 | 1.20 | 0.40 | 1.03 | 1.05 | 0.75 |
| Mean | 0.32 | 1.00 | 0.58 | 1.01 | 0.61 | 0.85 | 0.88 | 1.08 |
| SD | 0.09 | 0 | 0.25 | 0.73 | 0.38 | 0.68 | 0.57 | 0.94 |
| RSD | 27.2 | 0 | 42.4 | 72.1 | 61.8 | 79.5 | 63.9 | 86.3 |

**Table S2** Determination of the relative correction factor of unknown peak 1

| Reference peak | Relative correction factor *f_s_* at different concentrations (mg mL^-1^) | | | | | Mean | SD | RSD |
| --- | --- | --- | --- | --- | --- | --- | --- | --- |
|  | 5 | 10 | 15 | 20 | 25 |  |  |  |
| ChA | 0.68 | 0.74 | 0.70 | 0.69 | 0.73 | 0.71 | 0.03 | 3.67% |
| 3,5-DCQA | 1.81 | 1.88 | 1.76 | 1.67 | 1.43 | 1.71 | 0.18 | 10.34% |
| L-7G | 0.94 | 0.95 | 0.90 | 0.93 | 0.91 | 0.93 | 0.02 | 1.95% |
| 4,5-DCQA | 1.05 | 1.22 | 1.11 | 1.15 | 1.09 | 1.12 | 0.07 | 5.88% |
| A-7G | 0.66 | 0.72 | 0.67 | 0.67 | 0.67 | 0.68 | 0.02 | 3.52% |
| K-3R | 0.80 | 0.93 | 0.91 | 0.92 | 1.02 | 0.91 | 0.08 | 8.51% |

**Table S3** Determination of the relative correction factor of unknown peak 2

| Reference peak | Relative correction factor *f_s_* at different concentrations (mg mL^-1^) | | | | | Mean | SD | RSD |
| --- | --- | --- | --- | --- | --- | --- | --- | --- |
|  | 5 | 10 | 15 | 20 | 25 |  |  |  |
| ChA | 1.10 | 1.10 | 1.06 | 1.03 | 1.01 | 1.06 | 0.04 | 3.85% |
| 3,5-DCQA | 2.94 | 2.82 | 2.68 | 2.48 | 1.99 | 2.58 | 0.37 | 14.46% |
| L-7G | 1.53 | 1.42 | 1.38 | 1.38 | 1.27 | 1.39 | 0.09 | 6.69% |
| 4,5-DCQA | 1.70 | 1.82 | 1.70 | 1.71 | 1.52 | 1.69 | 0.11 | 6.48% |
| A-7G | 1.07 | 1.07 | 1.03 | 0.99 | 0.93 | 1.02 | 0.06 | 5.78% |
| K-3R | 1.31 | 1.38 | 1.39 | 1.38 | 1.41 | 1.37 | 0.04 | 2.75% |

**Table S4** Recovery of each analyte determined by standard addition method (n=3)

| Component | Known amount (mg g^-1^) | Added amount (mg g^-1^) | Measured amount (mg g^-1^) | Recovery rate (%) | Mean | RSD (%) |
| --- | --- | --- | --- | --- | --- | --- |
| ChA | 3.01 | 0.85 | 3.82 | 94.9% | 92.5% | 2.6% |
|  | 3.02 | 0.84 | 3.79 | 91.8% |  |  |
|  | 3.01 | 0.85 | 3.78 | 90.4% |  |  |
|  | 3.01 | 0.85 | 3.79 | 92.4% |  |  |
|  | 3.00 | 0.86 | 3.82 | 95.9% |  |  |
|  | 3.00 | 0.86 | 3.77 | 89.2% |  |  |
| 3,5-DCQA | 5.69 | 0.99 | 6.83 | 114.6% | 107.2% | 5.6% |
|  | 5.68 | 1.00 | 6.69 | 100.6% |  |  |
|  | 5.65 | 1.03 | 6.72 | 103.8% |  |  |
|  | 5.59 | 1.08 | 6.78 | 109.7% |  |  |
|  | 5.69 | 0.99 | 6.71 | 102.6% |  |  |
|  | 5.70 | 0.98 | 6.80 | 111.8% |  |  |
| L-7G | 12.79 | 2.51 | 15.35 | 101.6% | 105.4% | 2.4% |
|  | 12.81 | 2.50 | 15.40 | 103.7% |  |  |
|  | 12.89 | 2.42 | 15.47 | 106.5% |  |  |
|  | 12.74 | 2.56 | 15.49 | 107.2% |  |  |
|  | 12.65 | 2.64 | 15.51 | 108.2% |  |  |
|  | 12.76 | 2.54 | 15.43 | 105.1% |  |  |
| 4,5-DCQA | 5.92 | 1.68 | 7.45 | 91.1% | 93.0% | 3.4% |
|  | 5.91 | 1.69 | 7.44 | 90.4% |  |  |
|  | 5.95 | 1.65 | 7.53 | 96.2% |  |  |
|  | 5.92 | 1.68 | 7.53 | 96.1% |  |  |
|  | 5.89 | 1.71 | 7.53 | 95.7% |  |  |
|  | 5.91 | 1.69 | 7.40 | 88.3% |  |  |
| A-7G | 3.23 | 1.74 | 4.88 | 95.3% | 91.6% | 3.7% |
|  | 3.26 | 1.70 | 4.74 | 87.2% |  |  |
|  | 3.21 | 1.76 | 4.80 | 90.7% |  |  |
|  | 3.24 | 1.73 | 4.83 | 92.1% |  |  |
|  | 3.23 | 1.73 | 4.90 | 96.3% |  |  |
|  | 3.19 | 1.78 | 4.75 | 87.9% |  |  |
| K-3R | 13.59 | 1.48 | 15.28 | 113.8% | 110.2% | 6.2% |
|  | 13.53 | 1.54 | 15.12 | 102.8% |  |  |
|  | 13.59 | 1.43 | 15.22 | 109.7% |  |  |
|  | 13.61 | 1.46 | 15.21 | 109.2% |  |  |
|  | 13.59 | 1.56 | 15.38 | 120.4% |  |  |
|  | 13.59 | 1.49 | 15.16 | 105.7% |  |  |

**Table S5** Effect of extraction method on the contents of major components in CM ($\bar{x}$±SD, n=3)

| Extraction methods | Total polysaccharide (mg g^-1^) | Total phenolic acid (mg g^-1^) | Total flavonoid (mg g^-1^) |
| --- | --- | --- | --- |
| 50% ethanol reflux | 89.00±1.13^**^ | 15.89±0.12^**^ | 40.44±0.78^**^ |
| 50% ethanol ultrasound | 163.17±2.86 | 34.57±0.64 | 74.01±0.50 |
| Water Decoction | 99.84±1.42^**^ | 13.41±0.23^**^ | 37.41±0.71^**^ |

^**^*P* < 0.01, vs 50% ethanol ultrasound.

**Table S6** Effect of extraction solution on the contents of major components in CMs ($\bar{x}$±SD, n=3)

| Solvent | Total polysaccharide (mg g^-1^) | Total phenolic acid (mg g^-1^) | Total flavonoid (mg g^-1^) |
| --- | --- | --- | --- |
| Distilled Water | 145.93±8.94^**^ | 15.13±0.55^**^ | 33.06±1.77^**^ |
| 30% methanol | 163.19±10.05 | 27.13±0.21^#^ | 58.86±4.29^#^ |
| 50% methanol | 160.72±6.88^*^ | 28.68±0.34^**^ | 63.64±0.54^**^ |
| 70% methanol | 143.75±8.68^##^ | 28.62±0.64 | 59.9±1.34^##^ |
| 100% methanol | 100.41±2.31^##^ | 16.71±1.33^##^ | 43.99±3.38^##^ |
| 30% ethanol | 167.67±2.45 | 32.72±0.28^∆^ | 68.27±1.05^∆^ |
| 50% ethanol | 165.50±3.85 | 33.57±0.34 | 74.01±0.50 |
| 70% ethanol | 107.47±2.94^∆∆^ | 22.18±1.44^∆∆^ | 40.41±1.57^∆∆^ |
| 100% ethanol | 18.73±7.56^∆∆^ | 4.97±0.36^∆∆^ | 24.54±1.82^∆∆^ |

^**^*P* < 0.01 represents the *P* value between distilled water or 50% methanol and 50% ethanol; ^#^*P* < 0.01 or ^##^*P* < 0.01 represents the *P* value between other ratios of methanol and 50% methanol, respectively; ^∆^*P* < 0.01 or ^∆∆^*P* < 0.01 means the *P* value between other ratios of ethanol vs 50% ethanol, respectively.

**Table S7** Effects of extraction time on the contents of major components in CMs ($\bar{x}$±SD, n=3)

| Extraction time (min) | Total polysaccharide (mg g^-1^) | Total phenolic acid (mg g^-1^) | Total flavonoid (mg g^-1^) |
| --- | --- | --- | --- |
| 10 | 124.18±4.07^**^ | 26.27±0.72^**^ | 61.87±1.21^**^ |
| 15 | 131.79±2.05^**^ | 27.48±0.67^**^ | 69.27±2.32^**^ |
| 20 | 145.79±2.48^**^ | 29.46±0.88^*^ | 72.30±1.47^*^ |
| 30 | 161.09±2.24 | 31.53±1.28 | 74.73±1.13 |
| 40 | 125.41±8.99^**^ | 29.11±0.86^*^ | 75.86±1.12 |

^*^*P* < 0.05 or ^**^*P* < 0.01, vs the extraction time at 30 min.

**Table S8** Effects of liquid-to-solid ratio on the contents of major components in CMEs ($\bar{x}$±SD, n=3)

| Liquid-to-solid ratio (mL:g) | Total polysaccharide (mg g^-1^) | Total phenolic acid (mg g^-1^) | Total flavonoid (mg g^-1^) |
| --- | --- | --- | --- |
| 10:1 | 77.04±3.02^**^ | 16.44±0.88^**^ | 40.28±0.33^**^ |
| 20:1 | 160.26±7.01 | 33.58±0.64 | 70.31±4.55 |
| 30:1 | 161.63±5.64 | 32.04±1.61 | 67.54±2.15 |
| 40:1 | 163.02±10.21 | 32.21±1.32 | 68.17±3.21 |

^**^*P* < 0.01, vs the liquid-to- solid ratio at 20:1.
